# Sentiment and semantic analysis: Urban quality inference using machine learning algorithms

**DOI:** 10.1016/j.isci.2024.110192

**Published:** 2024-06-06

**Authors:** Emily Ho, Michelle Schneider, Sanjay Somanath, Yinan Yu, Liane Thuvander

**Affiliations:** 1 Department of Computer Science and Engineering, University of Gothenburg, Universitetsplatsen 1, 405 30 Gothenburg, Sweden; 2 Department of Computer Science and Engineering, Chalmers University of Technology, Chalmersplatsen 4, 412 96 Göteborg, Sweden; 3 Department of Architecture and Civil Engineering, Chalmers University of Technology, Chalmersplatsen 4, Göteborg 412 96, SE

**Keywords:** Artificial intelligence, Machine learning, Urban planning

## Abstract

Sustainable urban transformation requires comprehensive knowledge about the built environment, including people’s perceptions, use of sites, and wishes. Qualitative interviews are conducted to understand better people’s opinions about a specific topic or location. This study explores the automatization of the interview coding process by investigating how state-of-the-art natural language processing techniques classify sentiment and semantic orientation from interviews transcribed in Swedish. For the sentiment analysis, the Swedish bidirectional encoder representations from transformers (BERT) model KB-BERT was used to perform a multi-class classification task on a text sentence level into three different classes: positive, negative, and neutral. Named entity recognition (NER) and string search were used for the semantic analysis to perform multi-label classification to match domain-related topics to the sentence. The models were trained and evaluated on partially annotated datasets. The results demonstrate that the implemented deep learning techniques are a possible and promising solution to achieve the stated goal.

## Highlights

• Combining NER and string search enriches qualitative data interpretation
• NER can identify additional relevant labels, especially in the neighborhood class
• visual representation of semantic and sentiment relationships aids urban planners
• NLP techniques enhance understanding of residents’ perceptions through textual analysis

## Graphical abstract

**Figure:**
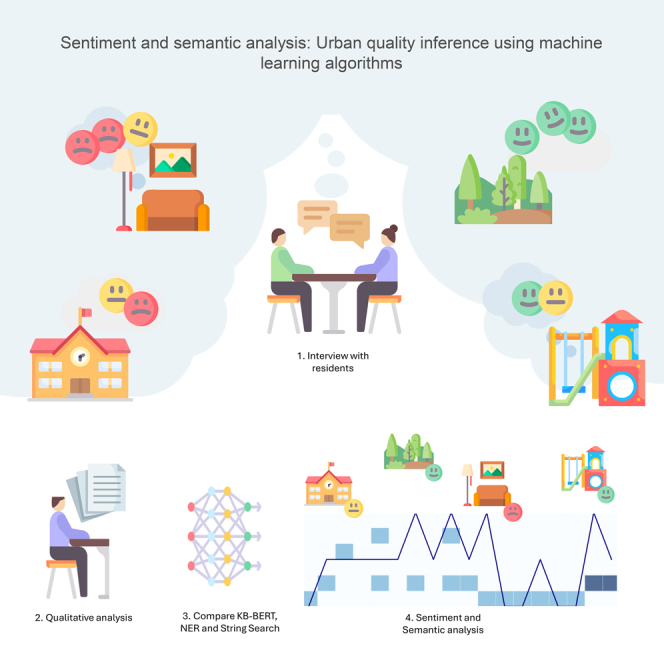

## Introduction

Urban planning can positively contribute to the health and well-being of the urban population, improving the living quality.^1^ For example, policies that regulate land use, transport, and green infrastructure are instrumental in improving health outcomes.^2^ More specifically, in Sweden, reducing car traffic in the city centers and building in favor of bikes and pedestrians has led to better health among the residents.^3^^,^^4^ To that end, a place’s environment and spatial planning are important in both policy development and their design solutions.^1^^,^^2^^,^^4^

One way to support the implementation of policies in creating urban spaces with high living qualities is to understand the resident’s perception of their neighborhood. This can be done through different qualitative research methods such as interviews, focus groups, and observations.^5^ Interviews are not only conducted by researchers studying urban qualities but also by local governments and urban planners to improve their understanding of how residents interact and feel about their local environment.

Interviews are usually recorded and later transcribed into text, the primary dataset for analysis. For the analysis, computer-assisted (or aided) qualitative data analysis software (CAQDAS) is used, which is a tool that assists with transcription analysis. Before the analysis, in the qualitative coding process of the transcribed text, descriptive or inferential labels are assigned manually to fragments of data in systematic order. However, performing such manual coding is labor-intensive and time-consuming, especially in the era of big data.^5^^,^^6^^,^^7^ To gain an overview of the data, visualizations such as tree maps and word clouds are used to show word frequencies. Yet, these methods do not consider the semantic relationships between people, places, and themes. Consequently, machine learning (ML) techniques have been leveraged to assist in the analysis of qualitative data^6^ to gain a deeper understanding of people’s attitudes and perceptions (sentiment) about a specific topic (semantic).

This study investigates how state-of-the-art natural language processing techniques classify sentiment and semantic orientation from interviews transcribed in Swedish and how these techniques can contribute to gaining an automated overview and coding process of the interview data. The overarching research question is:

How can state-of-the-art ML techniques be adjusted and applied to classify sentiment and semantic orientation from qualitative interviews, providing urban planners and researchers with an accurate overview of the data?

This paper focuses on extracting the sentiment and semantic context from Swedish transcribed interviews using ML techniques such as bidirectional encoder representations from transformers (BERT), named entity recognition (NER), and string search in urban planning. The connected sentiment and semantics are visualized as an interview summary diagram. The results are debated in the discussion and conclusion section.

### Background and related work

The following section introduces the domain of urban quality and the different approaches to its study in the literature. Urban quality research is a multidisciplinary topic that intersects with urban planning, psychology, and, more recently, computer science. In recent years, researchers have attempted to leverage developments in computational methods to enhance their ability to study urban qualities through techniques like text mining and other text-based research tools. Finally, we present the background on semantic and sentiment analysis and the developments in natural language processing.

#### Research on urban qualities

Urban quality is a multifaceted concept encompassing the degree of excellence or suitability of the urban environment for human well-being and development.^8^ Conceptually, perceived urban quality consists of three main components of cognition, affect, and behavior of the person perceiving an urban space; operationally, it is measured through inhabitants’ responses using empirical studies such as interviews and surveys.^8^ Fachrudin^9^ identified four broad indicators of urban quality: environmental quality, place quality in physical aspects, place quality in functional aspects, and safety of the urban space. Bonaiuto^8^ identified a basic issue in observational assessments of perceived urban quality as a lack of valid and reliable tools to operatively describe and measure constructs like satisfaction and quality. In principle, the literature identifies two approaches for data gathering, inductive and deductive approaches.^8^

Researchers^9^^,^^10^^,^^11^ have previously used deductive approaches using quantitative methods such as surveys and walkability studies to measure urban quality and sense of space. Fachrudin^9^ conducted a study of over 100 residents using questionnaire techniques and structural equation modeling to evaluate the influences of urban quality on a sense of place. The researchers found that functional, visual, and urban space experiences have an influence on the sense of place developed in the respondents. Kyttä^10^ used geographical information systems (GIS) techniques to conduct a web-based survey with over 3,000 participants to gather and analyze over 10,000 place experiences. Their findings revealed that location-based experiential information plays a valuable role in supporting evidence-based planning. The researchers found that urban qualities like density, green infrastructure, and distance traveled can have a significant impact on whether a place is perceived positively or negatively. Johansson^11^ investigated the associations between micro-level urban qualities and the experience of walking in neighborhoods through a study involving over 100 respondents. The results showed that perceived urban qualities such as aesthetics, upkeep, order, and greenery are identified as important and can serve as valuable input for urban design practices.

Inductive methods using interview techniques can yield deeper qualitative insights into understanding the different aspects of perceived urban quality. However, qualitative analysis can be tedious and time-consuming.^12^ Lauwers^13^ conducted 28 semi-structured interviews to describe the influences of the neighborhood on mental well-being. The interviews were transcribed verbatim and data analyzed was by a thematic analysis using the CAQDAS software Nvivo. Transcripts were read several times and a manual, inductive open coding was applied. The study provided detailed descriptions of physical neighborhood factors and social neighborhood factors that link to mental well-being. The interviews method was seen as advantageous to support participatory planning to detect complex interactions in the neighborhood environment. Marry^14^ studied everyday sounds perception in urban public spaces and sonic representations associated with urban typologies based on a qualitative survey, 18 focus groups*,* and 29 individual in-depth interviews, including 145 sonic mind maps to determine parameters which influence the perception of environmental sounds. Nvivo was used for the analysis of textual, iconographic, audio and video data and for occurrences of lemmatized vocabulary the Sphinx Lexica software was applied. The results indicate a link between visual parameters and the perception of sounds.

#### Semantic and sentiment analysis

In recent years, computational social sciences have emerged as a field of study to support the growing datasets, where machine learning (ML) and deep learning (DL) algorithms can be applied to assist in the identification and extraction of attitudes, opinions, and topics from interview transcripts.^5^^,^^6^^,^^7^ In particular, text classification methods have been leveraged to extract the sentiment and semantics of different texts. Text classification refers to the task of classifying written text, e.g., a word or a sentence, into predefined labels.^15^^,^^16^

**Figure 1:**
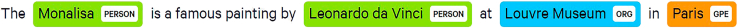
Example figure illustrating how NER models classify named entities using the displaCy visualizer In the figure, Monalisa and Leonardo da Vinci are classified as a person, Louvre Museum is recognized as an organization, and Paris is recognized as a geopolitical entity.

Sentiment analysis is a common example of multi-class text classification where written text is classified based on the expressed attitude toward a certain entity.^16^^,^^17^^,^^18^^,^^19^ These subjective expressions are often categorized into positive, negative, or neutral classes. In comparison, semantic analysis extracts the main topics that have been said in a given text. One of the most common techniques is text extraction, which includes entity extraction.^20^ Entity extraction attempts to identify all entities contained in a document. One method that comes into focus is NER, which is often used for semantic text extraction (Figure 1).

NER is a task within natural language processing (NLP) and semantic analysis where the objective is to detect and classify named entities in text.^21^ A named entity refers to a word or a series of words that identifies an item with similar attributes from a collection of other items, such as the name of an organization.^22^^,^^23^ For instance, *United Nations* is a named entity of the entity type *Organization*. NER is then the process of recognizing the names of the pre-defined semantic entities.^22^ Examples of named entity types are person, location, organization, dates, and times.^21^^,^^22^^,^^23^

Initial research within both sentiment and semantic analysis was first based solely on predetermined rules set by the model architect or language experts in this case, whereas the trend has moved on to using ML and now DL methods.^23^^,^^24^^,^^25^ Especially with the increasing amount of data generated, a major challenge with traditional ML models, such as naive Bayes and support vector machines, is that features are hand-crafted, where feature engineering and feature extraction are the most time-consuming processes. DL neural networks have thus been found to yield substantial improvements within NLP research.^26^ Compared to the traditional ML algorithms based on heavy feature engineering, the DL models have shown effectiveness with their ability to detect useful features through nonlinear processing automatically.^24^^,^^25^^,^^26^^,^^27^^,^^28^

Some popular DL models applied are convolutional neutral network (CNN), recursive neural network (RNN) including long short-term memory (LSTM), gated recurrent unit (GRU), bi-directional long short-term memory (Bi-LSTM), and transformer-based networks.^15^^,^^21^^,^^24^^,^^29^ As text classification tasks can also be considered sequential modeling tasks, recurrent neural networks (RNN) are more frequently used due to their ability to learn sequential associations, which is an important feature when dealing with semantic analysis of text.^15^^,^^30^ LSTM and Bi-LSTM models, which are based on RNN architectures, are capable of capturing word dependencies, taking into account the preceding and succeeding contexts in a text passage.^15^

Most recently, the state-of-the-art techniques used for sentiment analysis and the NER tasks are the transformer models.^29^^,^^31^ Transformer models process all words simultaneously rather than sequentially. Yet, it is able to ensure that the same word has different representations depending on its position in the sentence, and it can also learn the dependencies between words due to the self-attention mechanism.^29^ Using this approach, transformers have been shown to produce better results while at the same time being less time-consuming due to their ability to parallelize training.^28^^,^^29^ For NER tasks specifically, studies have shown that transformer-based models such as BERT, RoBERTa, and XLNet, can outperform non-transformer-based models such as RNN, CNN, LSTM, and other hybrid models as Bi-LSTM-CNN-CRF models.^21^

One specific kind of transformer is BERT, which stands for bi-directional encoder representations from transformers. BERT is a language model that was introduced by Devlin et al. in 2018.^32^ As the name implies, its purpose is to understand the meaning of words on both the left and right sides of the sentence. Its model is based on the multi-layer encoder architecture of the transformer model.

#### Use of ML to study urban quality

Researchers have previously used image based ML models to identify local architectural identity^33^ using automatic classification of morphological features in the images, perception of safety^34^ using convolutional neural nets on crowd sourced images and a combination of models^35^ to evaluate urban qualities like visual quality and visual continuity based on street view images using different deep convolutional neural networks and traditional ML techniques. Other than image data, researchers have explored the use of ML techniques on text data, particularly those from Quality of Life (QoL) surveys.^36^ used early forms of NLP such as term frequencies to turn qualitative data into quantitative data. More capable ML models made it possible to perform sentiment analysis as well as semantic analysis or topic modeling on text data. In recent years^37^ used data from online neighborhood reviews to perform semantic and sentiment analysis for different New York City neighborhoods. The researchers used an unsupervised Bayesian network model for semantic analysis called latent Dirichlet allocation (LDA), which was modeled on words like safe, crime, and police. Next, the researchers proposed a model to extract semantic orientations of reviews verified by their numerical rating associated with the text review. Similar research was conducted in Barcelona^38^ using regression models on Twitter data to extract public sentiments on urban environments and Dublin^39^ to evaluate opinions about urban green spaces based on TripAdvisor and Foursquare reviews using LDA. In both cases researchers found that tuning the models with context-specific keywords like “shopping”, “center”, and “history” improved model evaluation metrics like coherence values and model perplexity.

Addressing the challenges in observational assessments of urban quality research such as those posed by Bonaiuto,^8^ Thuvander^40^ conducted an interview study with 15 participants to investigate how they felt about their home and their surrounding urban areas, including meeting places in the neighborhoods or other places of interest. The researchers supplemented the interview techniques using paper maps to identify specific locations. The researchers evaluated ML-enabled tools available to conduct qualitative research, such as Atlas.ti and Nvivo in their study. The results showed that digital tools showed the potential to create nodes and clusters to visualize urban qualities and infer them in a systematic way, but at the same time, these methods had unexpected, limited potential regarding the automated clustering of urban qualities, visualization, and spatial integration. Hernandes^12^ explored different software tools to support researchers doing qualitative research but came to a similar conclusion that while these tools offer many resources to support the coding technique used in qualitative research, they do not address problems of analyzing a large number of research documents, simultaneously or automatically. To remedy this, the researchers conducted a feasibility study using an insight tool that used text mining and different visualization techniques to support qualitative research. The results showed that combining text mining and visualization with traditional qualitative research techniques can yield more benefits in qualitative analysis.

The application of ML in urban quality study has been applied to a wide range of data types and thematic areas within urban qualities research, such as architectural identity, safety, visual quality, perception, sentiment, and semantic orientations from textual data. Previous research on this topic has demonstrated the ability of using such models to turn qualitative data into quantifiable metrics. However, it is also clear that there are limitations regarding the automated clustering, visualization and spatial integration of these urban qualities. Moreover, the insights from previous research have not been applied to interview data.

## Results and discussion

This section provides an overview of the sentiment and semantic model results. Additionally, we generate a summary figure to provide a visual overview of the interview’s sentiments and semantics in a single image.

### Sentiment model

**Figure 2:**
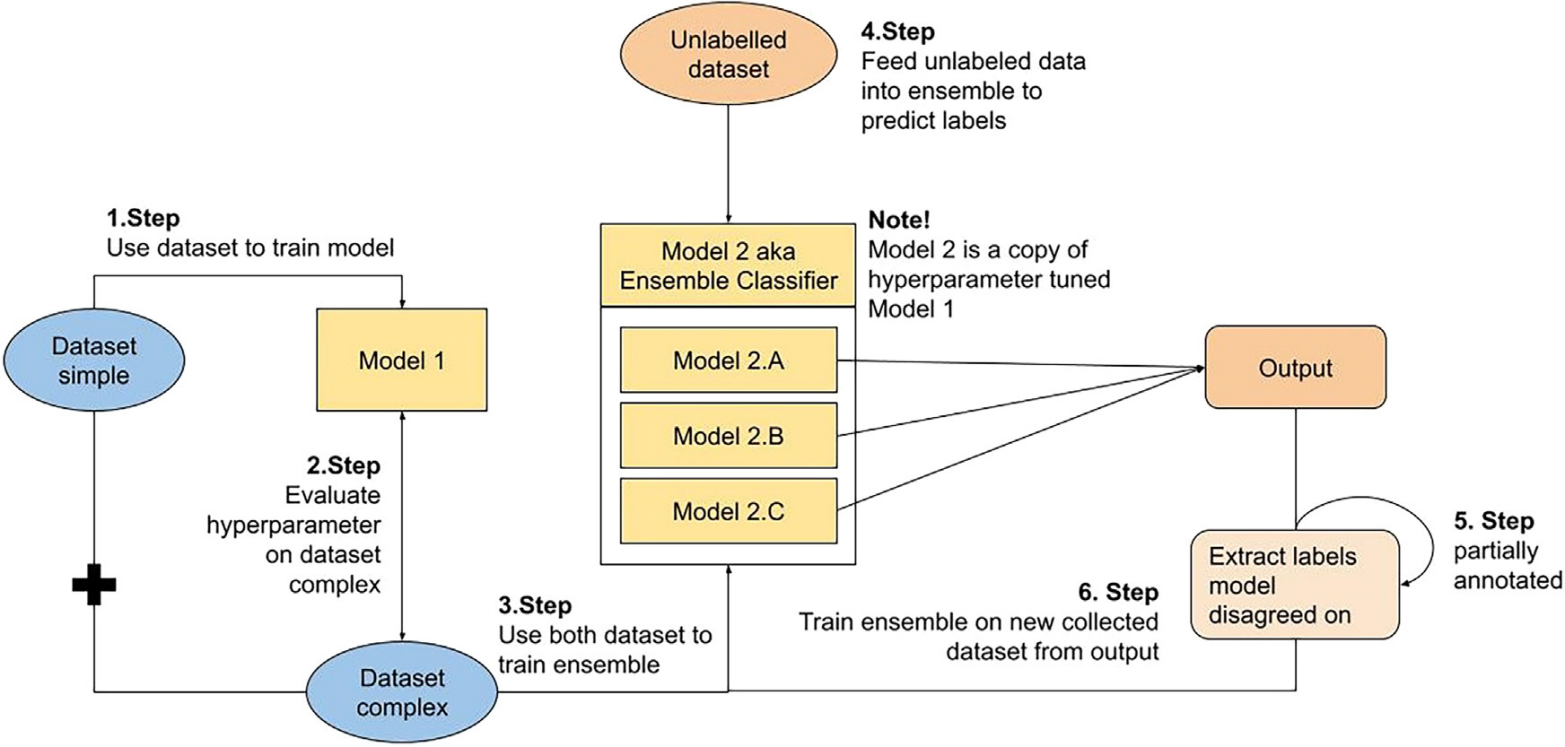
Figure illustrating the final ML model process

Table 1 presents the results of the model performance in terms of accuracy and weighted average and macro F1-score for the different sentiment models (see Figure 2). The transcript model performed the best with an accuracy of 0.85 and a macro F1-score of 0.80. The summary model, on the other hand, only had an accuracy and weighted average F1-score of 0.65. It can also be seen that the final transcript model performed worse after adding in the retrospective annotations, while the final summary model performed better with more annotations. These differences in performance could be attributed to the amount and class distribution of the datasets used for fine-tuning and evaluating the models.

**Table 1:** Evaluation metric comparison for sentiment model

Retrospective annotations	Without			With		
	Accuracy	Avg. F1	Macro F1	Accuracy	Avg. F1	Macro F1
Summary	0.6	0.61	0.59	**0.65**	**0.65**	0.64
Transcript	**0.87**	0.87	**0.82**	0.85	0.086	0.8

**Figure 3:**
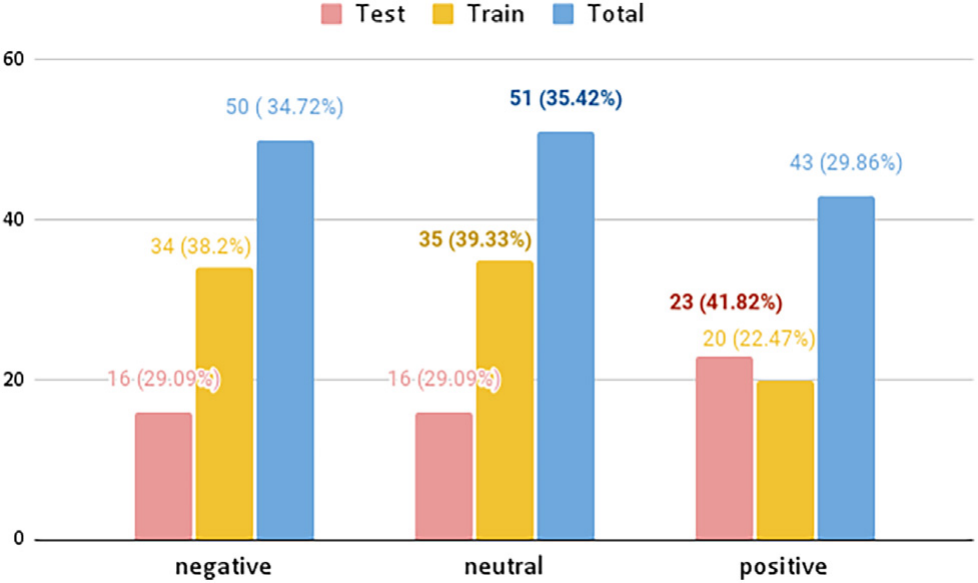
Distribution of data for sentiment analysis from interview summaries (left) and transcripts (right)

To further explain, the class distribution of negative, neutral, and positive of the annotated dataset can be seen to be significantly different for the summary and transcript models (see Tables 2 and 3). As illustrated in Figure 3, the summary dataset has almost a balanced distribution between the negative, neutral, and positive classes, while the transcript dataset mainly consists of the neutral classes. Therefore, the weighted average is looked at when evaluating the F1-scores for the summary, while the macro F1-score is inspected for transcripts. This is because macro F1 considers all classes equally important, while weighted average F1 considers the number of instances per class. As seen in Table 1, there is a negligible difference between the macro averaged F1-score and the weighted average F1-score for the summary datasets, and therefore, the class distribution does not have a big impact on the results. On the other hand, as seen in the transcript class in the same table, there is around a 4–7% difference between the macro and weighted average. This stresses that the model classifies sentences that belong to the classes with a larger distribution with higher confidence. In this case, the model is highly biased toward the neutral class.

**Table 2:** Overview of summary and transcript data

Interview	Annotated	Non-Annotated	Total
Summary	144	5,212	5,326
Transcript	561	42,606	43,167
Total	705	47,818	48,493
%	1.50%	98.60%	100.00%

(counts of sentences).

**Table 3:** Testing and training data distribution (counts)

Interview	Train	Test	Total
Summary	89	55	144
Transcript	475	86	561
Total	564	141	705

It can also be argued that with more training instances in the neutral class, the model will overfit toward the majority class and thus predict the instances as the majority. Consequently, more annotations may not always lead to better results. This also highlights a weakness of the DL models, which is a black box, and one cannot fully understand how they predict.

In supervised learning for ML and DL models, the quality and quantity of the target labels are also important to consider, as the models will learn based on the target labels provided. In this paper, for both the summary and transcript datasets, the annotated set does not even reach 3% of the total data. Moreover, the performance of the sentiment models is all based on two non-expert annotations. Therefore, the validity of the model can be questioned. On the other hand, in this use case, whether the model actually performs well or not depends on the final real-world use.

### Semantic model

Table 4 shows the results of the semantic models’ performance on the test set, where the labels found by the model were compared to the annotated target labels (see Table 5). Furthermore, Table 6 shows additional labels found by the model that were not marked in the annotated test set.

**Table 4:** Semantic results for both models in the categories summary (S) and transcript (T)

Model	Class	Acc S.	Predicted S.	Actual S.	Acc T.	Predicted T.	Actual T.
NER	nbr_scale	0.86	6	7	0.69	9	13
NER	org	0.50	1	2	**1.00**	1	1
SS	nbr_scale	**1.00**	7	7	0.77	10	13
SS	org	**1.00**	2	2	**1.00**	1	1
SS	nbr_sub	0.75	6	83	0.80	4	5
SS	room_scale	**1.00**	3	3	**1.00**	9	9
SS	unit_scale	0.95	19	20	0.93	13	14
SS	unit_sub	0.71	5	7	0.76	19	25

**Table 5:** Semantic annotated labels per semantic class

Interview	Summary	Transcript	Total
nbr_scale	7	13	20
org	2	1	3
nbr_sub	8	5	13
room_scale	3	9	12
unit_scale	20	14	34
unit_sub	7	25	32
Total	47	67	114

**Table 6:** Additional founded lables

Model	Class	Actual S.	Model S.	Actual T.	Model T.
NER	nbr_scale	7	**9**	13	**18**
NER	org	2	1	1	**2**
**NER**	**Total**	**9**	**10 (+1)**	**14**	**20 (+6)**
SS	nbr_scale	7	7	13	11
SS	org	2	**3**	1	**6**
SS	nbr_sub	8	7	5	**17**
SS	room_scale	3	**7**	9	4
SS	unit_scale	20	**30**	14	**28**
SS	unit_sub	7	**12**	25	24
**SS**	**Total**	**47**	**66 (+19)**	**67**	**90 (+23)**

When investigating the performance of the string search and NER model, the comparison has to be done within the classes *nbr_scale* and *org*. As seen in Table 4, NER has the highest accuracy value in the summaries is 0.86 in the class *nbr_scale* while string search achieves a value of 1. Also, within the class *org*, string search achieves better accuracy than NER. For the transcript interviews, both NER and string search got an accuracy value of 1 in the class “*org*”, while string search achieved a higher score in *nbr_scale* than NER. Possible reasons why NER performed worse than string search in both interview categories in the *nbr_scale* class could be due to the tokenization. A thorough investigation revealed that NER divides the word “*centrala Göteborg*” into two individual tokens, “*centrala*” and “*Göteborg*”. Since the given label is seen as a single token, the two would not match. As a result, it would be marked as a missing word and thus lower the accuracy.

Table 6 shows that both models have found more labels than were annotated. The bold numbers highlight the total and number of additional found labels. In summary, NER found +2 labels in the class *nbr_scale* while string search did not find any there, but it found +1 label in the class *org* whereas NER did not find any additional one. Similar performance can be seen on the transcript where NER is better in the class *nbr_scale* but worse in the class *org* compared to string search. A potential reason why NER performs worse in *org* could be that NER does not recognize domain-specific labels. For example, it was found that NER did not recognize the name of a Swedish housing association as an organization. It can be assumed that the word is not included in the vocabulary of NER, as in this case, it is a very specific housing organization in Gothenburg rather than being part of the general scope of a vocabulary.

### Visualization

As mentioned in the introduction, researchers and interviewers usually use tree maps and word clouds to visualize the interview data. However, this does not consider the semantic and sentiment relationship between people, places, and themes. With the predicted semantic and sentiment labels from the models, the results can be used to help investigate these relationships by transforming the results into visualization. It should be mentioned, however, that only string search is used for visualization at this moment because it can be applied to all semantic classes.

**Figure 4:**
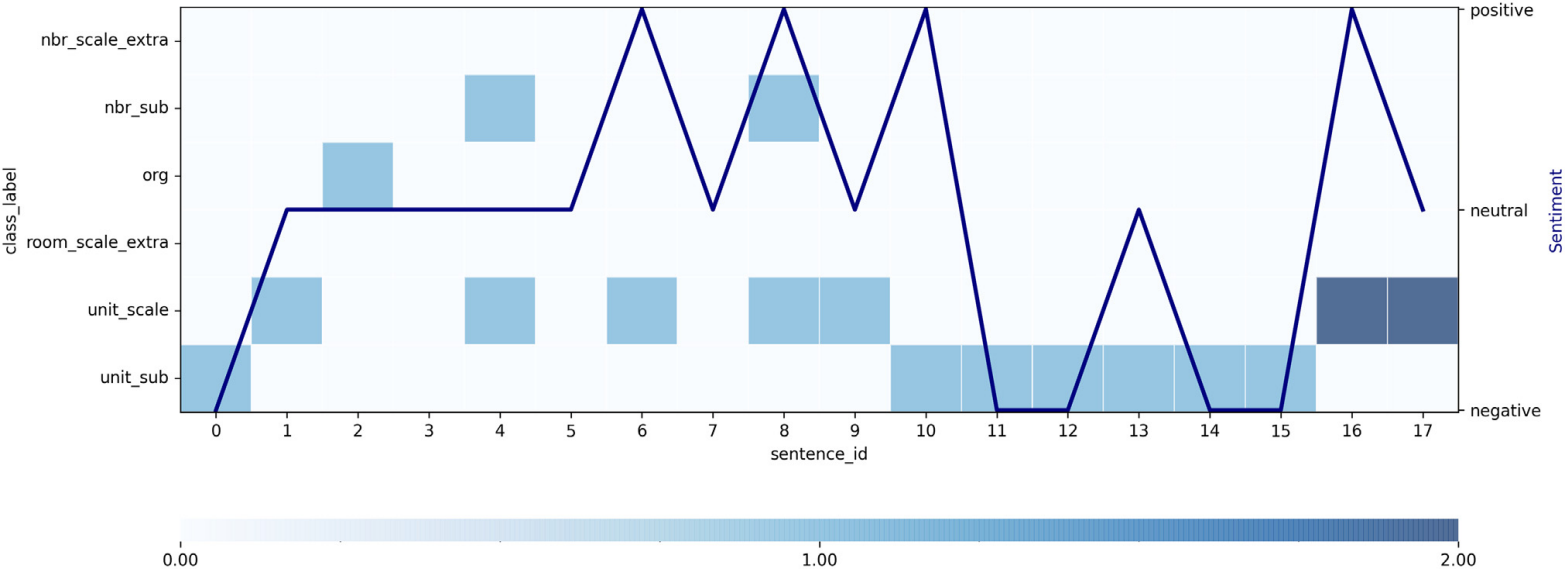
Overview visualization showing the sentence id along the horizontal axis, spatial classes identified through the semantic analysis on the left vertical axis and the sentiment on the right vertical axis The line plot shows the variation in sentiment as the interview progresses, and the heatmap shows the spatial class identified and its frequency.

In Figure 4, the final visualization for a summary interview can be seen. The semantic classes and the number of labels mentioned within each class and sentence are now shown as a heatmap, where the darker the color, the more instances of this class’ labels have been mentioned. The color bar at the bottom of the graph further describes this range of minimum and maximum number of labels found per class per sentence. The x axis represents the number of sentences within the interview, and the right y axis is the sentiment of positive, neutral, and negative. This visualization takes the difference between the number of labels into consideration as it is scaled based on the color.

In this particular graph, it can be seen that the beginning of the interview (sentences two to five) has neutral sentiments where each sentence contains words about different topics, such as unit_scale in sentence two or both unit_scale and nbr_sub in sentence four. A drastic change in the sentiment can be seen in the transition from sentence ten to eleven, where the sentiment changes from positive to negative. Here, both sentences have words that belong to the unit_sub class, which contains words that describe physical components within a unit such as *balkong* or *ventilation*. It might be interesting to investigate these two sentences in more detail by checking the actual text. Additionally, sentences 16 and 17 contain the most amount of words belonging to one class, which can be seen in the darker color. Two labels of the class *unit_scale* belong to both sentences. Also, sentence 16 has a positive sentiment, while sentence 17 has a neutral one.

### Limitations

Although extensive research is conducted within sentiment analysis, most of them have been modeled using English corpora.^18^^,^^24^ This results in a shortage of resources and tools, such as non-English datasets and benchmarks, making it difficult to build good sentiment classifiers for other languages such as Swedish.^18^^,^^25^ Therefore, the project was limited to models trained in Swedish. Nevertheless, other pre-trained BERT models on the Swedish corpus could be investigated and used for further exploration. Additionally, the sentiment data distribution showed in-balances. As illustrated in Figure 3, the summary dataset has almost a balanced distribution between the negative, neutral and positive classes, while the transcript dataset mainly consists of the neutral class at 66.49%, see Figure 3. Since the training and test dataset was based on whole documents, the project was limited to the given sentiment for each document and dataset.

### Conclusion

This study explored how state-of-the-art natural language processing techniques can classify sentiment and semantic orientation from interviews transcribed in Swedish, supporting an initial analysis of the interviews and providing an overview visualization. Our findings indicate that while each method has its strengths and limitations, their combined use enriches the interpretation of qualitative data, offering valuable insights for domain experts.

We found that the implemented deep learning techniques are a possible and promising solution to get a first overview of interview content. Although NER performs worse overall than string search in terms of accuracy for both summaries and transcripts for the given labels, it performs better than string search when finding additional labels for the *nbr_scale* class. Both models, however, complement each other well when finding additional information. It should also be noted that the NER has not been fine-tuned and, therefore, has potential for improvement, but this would require a dataset that has been fully annotated with the relevant NER tags of the respective domain.

Next, string search showed potential to serve as a good backbone for finding additional labels. Since the annotated dataset does not represent complete target values but merely serves as a sample value, the validity of the results is open to discussion. Yet, they provide informative insights into the models’ performance.

Finally, we found that using deep learning methods to complement qualitative research using text data is a possible and promising solution to extracting the sentiment and semantics of different texts. The combined visualization of the semantic and sentiment relationship on the sentence level provides a promising start for communicating the data content to the domain experts in the form of frequency and density of selected annotations.

The results of our research have implications for both qualitative researchers investigating urban quality and urban planners interested in getting a deeper insight into their study areas. Our method can help qualitative researchers in the initial analysis and coding process of the interview data. For urban planners, it can help them gain a deeper understanding of people’s attitudes and perceptions about a specific topic or location by identifying the main themes and sentiments expressed in the interviews.

Future research is needed to explore and develop these methods further. An important factor is that these deep learning models are heavily data-driven and need accurately annotated domain-specific data to reveal their full potential. Thus, forthcoming studies should involve domain experts from the field of urban planning or transformation in the annotation process, beginning with researchers, to ensure relevance and validation of the correctness of the interpretation of the results. In the next step, the spatial integration of the results should be aimed at.

For the next steps of this research, we plan on integrating the spatial dimension of the data by mapping the semantic and sentiment labels to the geographic locations mentioned in the interviews. This would help to get a deeper understanding of urban qualities in a neighborhood through a more intuitive way for urban quality researchers and practitioners.

## STAR★Methods

### Key resources table

**Table:** 

REAGENT or RESOURCE	SOURCE	IDENTIFIER
**Software and algorithms**		

KB-BERT	Hugging Face	BERT
Spacy	Spacy.io	NER
Scikit-learn	Scikit-learn.org	Machine Learning
Pandas	Pandas.pydata.org	Data Analysis
Numpy	Numpy.org	Scientific Computing

### Resource availability

#### Lead contact

Further information and requests for resources and data should be directed to and will be fulfilled by the lead contact, Sanjay Somanath (sanjay.somanath@chalmers.se).

#### Materials availability

Further information and requests for resources and data should be directed to and will be fulfilled by the lead contact, Sanjay Somanath (sanjay.somanath@chalmers.se).

#### Data and code availability

• The data used for this study consists of interview responses and can be shared upon request.
• Any code generated in this paper can be shared upon request.
• Any additional information required related to the data reported in this paper is available from the lead contact upon request.

### Experimental model and study participant details

The dataset for the text classification task is based on empirical data collected in a larger study of tenants’ experiences with housing renovation and their decisions to move from their homes (n = 447).^41^ The study was conducted in Gothenburg, Sweden, during the period of 2018 and 2021. In the above-mentioned study, researchers interviewed tenants who agreed to participate in the study and then transcribed the interviews to text to be further analysed and coded into different categories. Within this dataset, some items were in the format of interview transcripts and others in a written summary format.

Firstly, as sentiment and semantic classification were performed on a sentence level, each document was split into individual sentences. Then, the documents were cleaned of redundant information such as the date, time and place, and the initials and names of the interviewer and interviewee IDs were removed. Similarly, placeholders for laughter or inaudible words were removed. Summaries that included responses to a questionnaire at the end of the text were also deleted.

After cleaning and pre-processing the text (n=444), the final dataset containing transcripts (n=244) and summaries (n=200) was obtained, consisting of 43,110 and 5,354 individual sentences, respectively.

In addition, a list of eight semantic classes and their respective target labels was provided. The list consisted of eight semantic classes that included labels describing names of housing-related organisations, names of all neighbourhoods in Gothenburg, names of housing units, objects belonging to the neighbourhood, physical components within a unit, and rooms of a unit. As two classes of the list were found to be supplementary to existing ones, they were merged and cleaned up to obtain a comprehensive list with unique labels. There were finally 190 unique semantic labels grouped into six different semantic classes; housing-related organisations (*org*), neighbourhoods in Gothenburg (*nbr_scale*), housing units (*unit_scale*), objects belonging to the neighbourhood (*nbr_sub*), physical components within a unit (*unit_sub*), and rooms of a unit (*room_scale*). Furthermore, for both the semantic and sentiment analysis, the original dataset was unlabelled. The sentences did not have a target label for the topic discussed (such as neighbourhood, park, unit, floor) or a target label for the sentiment (if the opinion was negative, positive or neutral). Therefore, data had to be manually annotated to train and evaluate the models. For the sentiment, the sentences were labelled within one of the three classes: positive, negative or neutral. For the semantics, the test set was skimmed, and the most concise labels were extracted.

#### Final dataset

The final pre-processed dataset can be divided into annotated and non-annotated data, see Table 2. Overall, it was aimed to annotate around minimum of 1% for each interview category. Since the whole documents were used to grasp the whole context, ten interviews consisting of a total of 705 sentences were annotated, four from the category summaries and six from the category transcripts, where 561 (79.60%) sentences belong to transcripts and 144 (20.40%) to summaries. For both training and evaluating steps, the models used only the annotated dataset. As such, the labelled set was split into a training and test set, as seen in Table 3. The annotated sentiment distribution of the different interview formats and datasets can be seen in Figure 3.

The annotated training dataset was also separated into two different sets, ${\text{Dataset}}_{\text{simple}}$ and ${\text{Dataset}}_{\text{complex}}$, to train and validate the final sentiment model in the later process. The ${\text{Dataset}}_{\text{simple}}$ includes sentences that contained annotator opinion matches, while the ${\text{Dataset}}_{\text{complex}}$ consists only of those sentences where the annotators initially disagreed but agreed on a sentiment class in a later discussion. Out of the total 705 annotated sentences, 595 sentences (84.40%) can be found in ${\text{Dataset}}_{\text{simple}}$, while a disagreement on 110 sentences (15.60%) belongs to ${\text{Dataset}}_{\text{complex}}$.

As the semantic models were unsupervised and did not require any training, the annotations were applied only to the test set. That is, only 55 summary and 86 transcript sentences were labelled. It should be mentioned that each sentence can be assigned to none or more than one label from a different or the same class. Table 6 illustrates the distribution of annotated labels per semantic class for both the summary and the transcript test sets.

### Method details

The following section outlines the methods used to train the sentiment analysis model, the semantic model and the final inference model.

#### Sentiment model

A multi-class text classification model for sentiment analysis was implemented using the language-specific pre-trained BERT model for Swedish (”KB-BERT”, specifically bert-base-swedish-cased (v1)) which was developed by KBLab at the National Library of Sweden (KB)^42^ and fine-tuned to suit the domain.

The last hidden layer of the KB-BERT model was extracted, and a single-hidden layer feed-forward neural network was implemented as the sentiment classifier. This model was implemented for both the summary and the transcript format.

Since this approach follows a semi-supervised methodology and only 1.5% of the data is annotated, additional approaches had to be applied to ensure an accurate outcome. For this reason, a voting ensemble classifier was created with the aim of better performance compared to a single classifier. As the name suggests, a voting ensemble chooses the label based on the outcome of the soft majority of classifier predictions, i.e. each model predicts a probability for each class. These are accumulated and the highest value in one of the classes is selected. Therefore, the complete sentiment model consists of three pre-trained KB-BERT models that were fine-tuned according to the dataset provided earlier.

First, the KB-BERT model was trained using the ${\text{Dataset}}_{\text{simple}}$, and the hyperparameters, batch size and learning rate were fine-tuned based on the ${\text{Dataset}}_{\text{complex}}$. These two datasets were then used to train each model within the ensemble classifier again. To further improve the reliability, it was decided to train the ensemble classifier again on another retrospectively annotated dataset. This time, the dataset was created from the unlabelled dataset, where the ensemble was first used to predict the full unlabelled set. For the predicted sentiment of sentences on which the three classifiers did not agree, the sentences were annotated manually and fed into the models. 301 and 401 sentences were annotated for the Summary and Transcript category was annotated, respectively. Thereby, in total, 1407 annotated sentences were used for training and evaluation, which is around 2.9% of the full dataset.

The different metrics used for evaluation were precision, recall, and accuracy. Also, the F1-score, for the summaries, a weighted average of the F1-score was used, while for the transcripts, a macro F1-score was applied for evaluation.

#### Semantic model

To discover the semantics, the Swedish BERT model fine-tuned for NER (*bert-base-swedish-cased-ner*), also developed by KBLAB^42^ and a naive String Search algorithm was used. From the given pre-trained NER tags, only the entity types location *(LOC)* and organisation *(ORG)* are of interest for this use case.

The String Search, on the other hand, followed a string matching algorithm to find if a string is found within a larger piece of string or text. This work was carried out separately for each semantic class and thus a total of six times. The text input and the labels for four out of six classes were lemmatised in order to capture all the different inflections of the words. First, to capture the labels of *’unit_sub’,’unit_scale’,’nbr_sub’*, and *’room_scale’* in the text, both the labels and the input text had to be lemmatised in order to capture all the different inflections of the words. For the two other classes (*’org’, ’nbr_scale’*), the original label and text were compared as the actual names of organisations and neighbourhoods are of interest.

In order to evaluate the performance of the semantic models, the given partially annotated test dataset was used as the target value. Due to this, only accuracy is considered in this thematic context.

#### Final inference model

To clarify the application use of this model for predicting the sentiment and semantics of new interview documents, only the ensemble classifier was used for the sentiment prediction, while both String Search and NER were used for semantic prediction. These predicted labels can then be used for visualisation, as will be discussed later.

In recent years, large language models (LLMs) such as ChatGPT have been widely adopted. These models are capable of not only engaging in human-like conversations but also performing tasks such as data analysis, content generation, language translation, and educational assistance. LLMs’ capabilities of analyzing interview data have been explored in the literature.^43^ Some researchers argued that LLMs can introduce serious bias when summarizing interview data, and therefore it is inevitable to annotate data for validation purposes. Given this necessity, it is probably preferable to train a domain specific model using that annotated data.^44^ Another consideration of choosing a smaller model over an LLM is that smaller models, due to their reduced scale, consume significantly lower computational resources for both training and inference. This efficiency extends to other aspects as well, including lower carbon footprints, making them more environmentally sustainable and accessible for deployment. Another notable advantage of smaller models is their capacity for domain-specific adaptations. While LLMs are generally pre-trained on vast and diverse datasets, they might not be as effective in handling specialized or niche domains typical in interview data. Smaller models, on the other hand, can be fine-tuned to adapt more effectively to specific annotations and tasks. Privacy and compliance with regulations such as the General Data Protection Regulation (GDPR) also play a crucial role in the preference for smaller models. The scale and complexity of LLMs can make it challenging to ensure complete adherence to privacy norms and regulatory standards. This issue is compounded by the fact that LLMs are often hosted as proprietary systems.

In such settings, uploading sensitive interview data can be problematic, as it may raise concerns about data security and privacy. Moreover, smaller models are often easier to understand and manage, making them a preferred choice for researchers who value ease of use and transparency in their analysis. This simplicity also aids in version control and maintenance, as managing and updating smaller models is typically less cumbersome than dealing with large-scale LLMs. However, one restrictive factor of small language models is that they are typically only capable of handling one specific language. Based on these considerations, we chose the Swedish model KB-BERT in our study.
